# Culture-Dependent Bioprospecting of Bacterial Isolates From the Canadian High Arctic Displaying Antibacterial Activity

**DOI:** 10.3389/fmicb.2019.01836

**Published:** 2019-08-09

**Authors:** Evangelos Marcolefas, Tiffany Leung, Mira Okshevsky, Geoffrey McKay, Emma Hignett, Jérémie Hamel, Gabriela Aguirre, Olivia Blenner-Hassett, Brian Boyle, Roger C. Lévesque, Dao Nguyen, Samantha Gruenheid, Lyle Whyte

**Affiliations:** ^1^Department of Natural Resource Sciences, McGill University, Sainte-Anne-de-Bellevue, QC, Canada; ^2^Department of Microbiology and Immunology, McGill University, Montreal, QC, Canada; ^3^Institute for Integrative Systems Biology, Université Laval, Quebec City, QC, Canada

**Keywords:** Arctic, bioprospecting, antibiotics, secondary metabolites, microbial cultivation

## Abstract

The goal of this study was to isolate, screen, and characterize Arctic microbial isolates from Expedition Fjord, Axel Heiberg Island, Nunavut, Canada capable of inhibiting the growth of foodborne and clinically relevant pathogens. Arctic bacteria were isolated from twelve different high Arctic habitats pertaining to active layer permafrost soil, saline spring sediments, lake sediments, and endoliths. This was achieved using (1) the cryo-iPlate, an innovative *in situ* cultivation device within active layer permafrost soil and (2) bulk plating of Arctic samples by undergraduate students that applied standard culturing methods. To mitigate the possibility of identifying isolates with already-known antibacterial activities, a cell-based dereplication platform was used. Ten out of the twelve Arctic habitats tested were found to yield cold-adapted isolates with antibacterial activity. Eight cold-adapted Arctic isolates were identified with the ability to inhibit the entire dereplication platform, suggesting the possibility of new mechanisms of action. Two promising isolates, initially cultured from perennial saline spring sediments and from active layer permafrost soil (*Paenibacillus* sp. GHS.8.NWYW.5 and *Pseudomonas* sp. AALPS.10.MNAAK.13, respectively), displayed antibacterial activity against foodborne and clinically relevant pathogens. *Paenibacillus* sp. GHS.8.NWYW.5 was capable of inhibiting methicillin resistant and susceptible *Staphylococcus aureus* (MRSA and MSSA), *Listeria monocytogenes*, *Salmonella enterica* and *Escherichia coli* O157:H7. *Pseudomonas* sp. AALPS.10.MNAAK.13 was observed to have antagonistic activity against MRSA, MSSA, *Acinetobacter baumanii*, *Enterococcus faecium*, and *Enterococcus faecalis*. After whole genome sequencing and mining, the genome of *Paenibacillus* sp. GHS.8.NWYW.5 was found to contain seven putative secondary metabolite biosynthetic gene clusters that displayed low homology (<50% coverage, <30% identity, and e-values > 0) to clusters identified within the genome of the type strain pertaining to the same species. These findings suggest that cold-adapted Arctic microbes may be a promising source of novel secondary metabolites for potential use in both industrial and medical settings.

## Introduction

The rise of antibiotic resistance is one of the most urgent challenges the world currently faces. Antimicrobial resistance has steadily increased in clinical settings (Bitnun and Yeh, 2018). We are on the cusp of returning to a pre-antibiotic world in which common infections and minor injuries will once again become deadly (Laxminarayan et al., 2013; Ferri et al., 2017; Pawar et al., 2017). Simultaneously, natural product discovery efforts on the part of the pharmaceutical industry have largely dwindled since the end of the 20th century (Baker et al., 2007). Given that the current arsenal of effective antibiotics is decreasing, innovative discovery workflows for the identification of novel antibiotics are needed.

*Enterococcus faecium*, *Staphylococcus aureus*, *Klebsiella pneumoniae*, *Acinetobacter baumannii*, *Pseudomonas aeruginosa*, and *Enterococcus* species (ESKAPE) are recognized by the Infectious Disease Society of America as the bacteria posing the most significant risk to public health in the United States (Boucher et al., 2009). The ESKAPE pathogens are responsible for the majority of nosocomial infections in the United States, with an estimated 722,000 infections acquired in 2011 (Magill et al., 2014). Of particular concern are the increasing levels of antibiotic resistance occurring in these organisms, especially methicillin-resistant *S. aureus* (MRSA), vancomycin-resistant *E. faecium*, and fluoroquinolone-resistant *P. aeruginosa* (National Nosocomial Infections Surveillance System, 2004). MRSA infections are now responsible for more deaths in U.S. hospitals than HIV/AIDS and tuberculosis combined (Klevens et al., 2006; Boucher and Corey, 2008).

Many of the antibiotics currently in use are secondary metabolite natural products from soil bacteria, particularly from the *Streptomyces* genus. Since the pioneering experiments of Selman Waksman in the 1940’s, this resource has been extensively studied (Waksman and Woodruff, 1940; Schatz et al., 1944). New approaches or modification to existing methods may be necessary to increase the probability of finding novel compounds. Once an isolate with antibiotic activity is identified, a “dereplication” procedure is required to avoid the re-discovery of already-known antibiotics. Compound extraction, purification and biochemical analyses are costly and technically challenging. Instead, we have employed a cell-based dereplication platform (Cox et al., 2017), in which isolates are tested for inhibitory activity against a panel of *Escherichia coli* strains expressing specific antibiotic resistance genes. The presence of inhibitory activity against all dereplication strains that comprise the Antibiotic Resistance Platform (ARP) suggests that the isolate produces antimicrobial secondary metabolite(s) with a potentially novel mechanism of action. As an additional dereplication measure, genomic sequencing and *in silico* genome mining could be used to prioritize isolates for downstream testing. Microbial genomic sequences could be mined *in silico* to detect secondary metabolite biosynthetic gene clusters (BGCs) using open source web-based pipelines such as the antibiotics and secondary metabolite analysis shell (antiSMASH) (Blin et al., 2019). Once microbial genomes have been mined, isolates can be prioritized on the basis of low BGC sequence homology to known clusters (Medema et al., 2015).

In the search for new antibiotics, interest has been growing in underexplored environments such as marine systems (Jang et al., 2013; Machado et al., 2015) and the deep biosphere (Orsi et al., 2013). The Canadian high Arctic is characterized by extreme environmental conditions such as high salt and prolonged subzero temperatures. The unique ecological niches (i.e., hypersaline springs, permafrost and endoliths) of the high Arctic harbor diverse microbial communities which remain largely unexplored (Perreault et al., 2007, 2008; Steven et al., 2007; Lay et al., 2013). Recent macro- and microdiversity studies have revealed that Arctic microbiomes do not always follow the latitudinal diversity paradigm, which states that diversity decreases towards the poles (Gregory et al., 2019). Arctic environments have been observed to be a cradle for microbial diversity (Gregory et al., 2019). Due to the rich microbial diversity and unique selective pressures experienced by the Arctic microbiome, we hypothesize that this extreme environment has potential for harboring novel antibacterial secondary metabolites. Previous bioprospecting studies conducted in polar cryohabitats have successfully identified microbial isolates expressing new natural products and encoding unknown secondary metabolite BGCs (Mitova et al., 2005; Tedesco et al., 2016; Dhaneesha et al., 2017). One such example includes the discovery of *Streptomyces artemisiae* MCCB 248 isolated from sediments in the Arctic fjord Kongsfjorden. This isolate was shown to produce secondary metabolites with anticancer properties and was found to encode unique polyketide synthetase (PKS) and non-ribosomal peptide (NRP) genes (Dhaneesha et al., 2017). Isolating secondary-metabolite-producing microbes from Arctic environments has the additional benefit of yielding strains with inhibitory activity at cold temperatures. Cold-active antimicrobial enzymes, such as cold-active alkaline phosphatases with antibiofilm activity, represent appealing biopreservatives for food processing industries since they can extend the shelf life of refrigerated consumables (Balabanova et al., 2017).

Identifying and isolating new antibacterial secondary metabolites often depends on cell-based screening of cultivable strains. Co-culture assays are known to activate silent BGCs in antibiotic-producing strains that would not be expressed in pure culture (Zarins-Tutt et al., 2016). Unfortunately, only a small percentage of all microbial species can be isolated using classic cultivation techniques (Winterberg, 1898; Ward et al., 1990). Innovative cultivation techniques are expanding the portion of cultivable isolates that could be phenotypically screened using cell-based assays (Vartoukian et al., 2010). The efficacy of such *in situ* cultivation techniques for drug discovery is illustrated by the recent discovery of the antibiotic Teixobactin, derived from an ichip isolate (Ling et al., 2015). Based on the initial design of the ichip, the cryo-iPlate prototype designed and implemented by Goordial et al. (2017) was the first *in situ* cultivation device deployed in the Arctic. Here, a re-designed and updated version of the cryo-iPlate is described for the first time and is used to isolate Arctic microorganisms for antibacterial screening. In addition to sampling unique ecological habitats and implementing the new cryo-iPlate cultivation device, the bioprospecting workflow described here uses a crowd-sourced screening approach first developed by Jo Handelsman at Yale University in 2012, which harnesses the manpower of undergraduate microbiology teaching labs to screen bacterial isolates for antibiotic activity (Davis et al., 2017).

**FIGURE 1:**
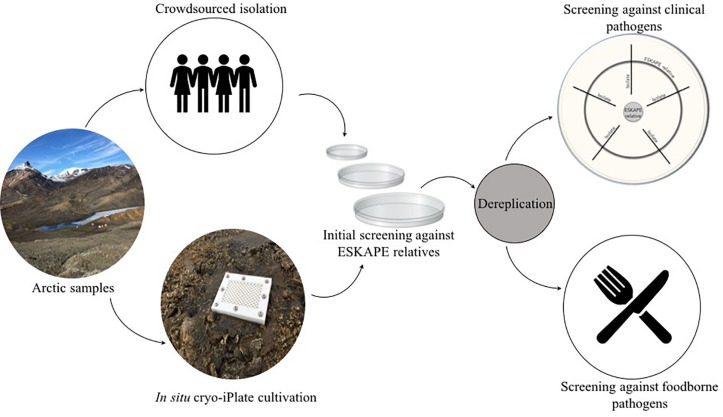
Overview of the Arctic bioprospecting workflow.

Here, we employed two cultivation methods to isolate and then screen bacteria derived from Canadian high Arctic habitats for antibacterial activities (Figure 1). Arctic isolates were screened for antibiotic activity against a collection of both foodborne and clinical pathogens. Cold-adapted antibiotic-producing bacteria were derived from Arctic permafrost, saline spring sediments, and cryptoendoliths suggesting that high Arctic environments could be a potentially untapped source of novel antibacterial secondary metabolites.

## Materials and Methods

### Sampling Arctic Microbiomes

The various soil and rock samples used in this study were collected within the vicinity of the McGill Arctic Research Station (MARS) on Axel Heiberg Island, Nunavut, in the Canadian high Arctic. The sediment, soil and rock samples were collected for crowdsourced bulk plating whereas the *in situ* cryo-iPlate cultivation approach was only applied to active layer permafrost soil (described below). Sediments from spring outflow channels and soil in this study were collected with an ethanol-sterilized spatula used to exhume the first 15 cm of soil/sediment horizons. Rocks with visible lithic microbial biomass were sampled by breaking large rocks into smaller pieces using an ethanol-sterilized hammer and chisel. All rock and soil samples were collected in sterile WhirlPak bags and stored in coolers kept at 5°C until returned to McGill University. Upon return to the laboratory, all samples were stored at 5°C.

### Cryo-iPlate Procedures

The cryo-iPlate design was based on the Nichols et al. (2010) ichip and the Goordial et al. (2017) cryo-iPlate prototype (Nichols et al., 2010; Goordial et al., 2017). It was designed using the Rhino 6 software and 3D-printed at Fablab Inc., Montreal, using durable PC-ISO polycarbonate plastic. Previous prototypes of the cryo-iPlate such as the one described in Goordial et al. (2017), consisted of empty pipette boxes that were not as durable in the field. The dimensions of the improved version of the cryo-iPlate described herein are 15 cm by 12 cm. It features 160 wells (as opposed to 96 in previous prototypes) that are 0.5 cm wide and deep. The wells of the central plate were filled with 2% w/v gellan gum (Alfa Aesar) prior to being deployed in the field. Based on previous studies of total microbial counts in active layer permafrost at the MARS study site, dilutions of the soil were made in the field using sterile water (Wilhelm et al., 2011). This device was only applied to active layer permafrost. Each individual well of the central plate was inoculated in the field with 10 μl of diluted soil containing ∼1–10 microbial cells. The top and bottom layers were overlaid with sterile semi-permeable 0.03 μm-pore-size polycarbonate membranes (Whatman^TM^, GE Healthcare Life Sciences) and were glued using a silicon adhesive (DuPont). The assembly of three plates was then screwed together using eight stainless steel 10–24 screws and left to incubate *in situ*. After incubating in the field for 10 days, the cryo-iPlate was collected in a large sterile Whirl-Pak sampling bag along with the soil in which it was incubating. The assembly was stored in a cooler at 5°C during transport back to McGill University where it was subsequently incubated *ex situ* at 5°C for 3 months before being disassembled for subculturing. After disassembling the cryo-iPlates, well contents from the central plate were pushed into individual Eppendorf tubes containing 0.1% w/v pyrophosphate using sterile 1000 μl pipette tips (Diamed Canada). Eppendorf tubes containing well contents from the cryo-iPlate in buffer solution were vortexed for 30 s and 100 μl of the solution was used to inoculate plates of 1/2 Reasoner’s 2A (R2A) broth media solidified with 2% gellan gum (Alfa Aesar). The plates were incubated at room temperature for 2 weeks. Morphologically distinct colonies (selected based on distinct colony form, elevation, margin, size, texture and color) that grew on the 1/2 R2A plates were subcultured three times to attain clonal populations before being screened against the ESKAPE relative strains.

### Crowdsourced Screening of Isolates Against ESKAPE Pathogen Relatives

**FIGURE 2:**
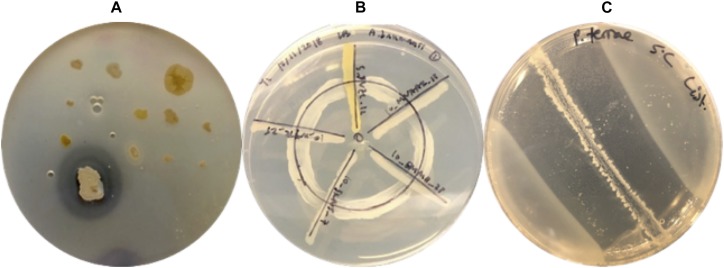
Three different co-culture techniques used to screen Arctic isolates for antibacterial activity. **(A)** Spread-patch assay applied by the undergraduate crowdsourcing initiative to screen Arctic isolates against ESKAPE relatives. The image above features an Arctic bacterial isolate from the undergraduate crowdsourcing initiative exhibiting a zone of clearance against a lawn of an ESKAPE relative tester strain. **(B)** Wagon wheel assay used to dereplicate and screen Arctic isolates against clinically relevant pathogens. The image above displays five Arctic isolates: GHCE.5.JVZL.12, AALPS.10.MNAAK.13, AALPS.10.EMMH.23, AALPS.10.JKNJ.7, MAL.10.WYTK.25 (in clockwise order) tested against the tester pathogen *A. baumanii*. **(C)** Overlay assay used to screen Arctic isolates against foodborne pathogens. The image above displays the positive control strain *Paenibacillus terrae* NRRL B-30644 inhibiting the growth of *L. monocytogenes* at 5***°***C.

The twelve sample types described in Table 1 were processed by students in the Introduction to Microbiology Laboratory Course at McGill University in the following manner. One gram of sample was serially diluted in sterile water to 10^–5^, and 100 μL of each dilution were spread onto lysogeny broth (LB) plates solidified with gellan, tryptic soy agar (TSA) (10% or 0.1%), potato dextrose agar or sheep blood agar. Each student pair participating in the course was provided with one of the Arctic samples and was allowed to select one of the aforementioned culturing media to increase the diversity of isolates obtained. Plates were incubated for 1 week at room temperature and morphologically distinct colonies were picked (selected based on colony form, elevation, margin, size, texture and color) and re-streaked to produce libraries of clonal populations. Isolates were screened against ESKAPE pathogen relatives (Supplementary Table S1) using the spread-patch technique (Figure 2A). Since antibiotic activity screening is performed in teaching labs, isolates were tested against non-pathogenic relatives of high priority threat pathogens. A diluted suspension of tester bacteria was evenly spread across on agar plate, and the Arctic isolates spotted on top. Plates were incubated for 1 week at room temperature and observed for a zone of pathogen inhibition surrounding isolates. All isolates that produced a zone of inhibition were given a name and stored as glycerol freezer stocks and selected for dereplication (Figure 1). If isolates were surrounded by a visually discernible zone of clearance devoid of any tester strain growth, then they were deemed to have positive antibacterial activity (Figure 2A).

**TABLE 1:** Arctic samples collected from Expedition Fiord, Axel Heiberg Island, Nunavut, used as source material for bioprospecting of antibiotic-producing strains.

**Sample type**	**Source location**	**Geographic coordinates**	**Habitat**
Active Layer Permafrost	MARS station Expedition Fiord	(79.412872, – 90.740343)	Trough soil from polygonal tundra terrain
			Surface soil from polygonal tundra terrain
			Arid soil
			Active layer tundra permafrost
	Gypsum Hill, Expedition Fiord	(79.403906, – 90.732321)	Hummock soil
Sediments	Junction Diapir (Lost Hammer)	(79.076755, –90.195299)	Perennial hypersaline (22–23% salt) spring outflow channel sediment (Pollard et al., 2009)
	Color Lake, Expedition Fiord	(79.416300, – 90.764081)	Acidic (pH ~3.6) freshwater lake sediment (Johannesson and Lyons, 1995)
			Acidic (pH ~3.6) freshwater lake sediment covered with microbial mat (Johannesson and Lyons, 1995)
	Gypsum Hill, Expedition Fiord	(79.403906, – 90.732321)	Perennial saline (7.5–15.8% salt) spring sediment (Perreault et al., 2007)
	White Glacier, Expedition Fiord	(79.431663, – 90.647073)	Sediment from glacial terminus moraines
Lithic Communities	Gypsum Hill, Expedition Fiord	(79.412151, – 90.740644)	Gypsum cryptoendolith
	Junction Diapir (Lost Hammer)	(79.076755, – 90.195299)	Unknown rock cryptoendolith

### Taxonomic Identification of Isolates

Isolates from the cryo-iPlate and from the crowdsourced bulk soil plating that displayed zones of inhibition against at least one of the ESKAPE relatives were selected for 16S rRNA gene sequencing. Colony PCR was first conducted to amplify the 16S rRNA gene using the 27F (forward) bacterial primer: 5′-AGAGTTACCTTGTTACGACTT-3′ and the 1492R (reverse) bacterial primer: 5′-GGTTACCTTGTTACGACTT-3′. The 16S rDNA amplification PCR reaction cycling program consisted of: (1) 95°C for 7 min, (2) 94°C for 45 s, (3) 55°C for 45 s, (4) 72°C for 1 min, (where steps 2 to 4 were repeated 30 times), (5) 72°C for 10 min. PCR products from the cryo-iPlate and from the bulk soil plating were sent for Sanger sequencing at the Plateforme d’Analyses Genomiques sequencing center at Laval University and the McGill University and at the Génome Québec Innovation Centre (respectively). All generated sequences were manually curated using 4Peaks software^1^ where sequence segments with an average Q > 40 were used as queries against the EzBioCloud database for taxonomical identification (Yoon et al., 2017).

### Dereplication Platform

Isolates that demonstrated antibacterial activity against at least one ESKAPE relative were tested against a dereplication platform described in Supplementary Table S2, and provided by the Wright lab at McMaster University (Cox et al., 2017). This dereplication platform consists of two parental strains: Wild type *E. coli* BW25113, and *E. coli* Δ*bamB* Δ*tolC* BW25113 harboring mutations to increase outer membrane permeability and decrease efflux pump activity, thereby increasing its susceptibility to antibiotics. In all cases except the *fluB* mutant, the Wright lab transformed these parent strains with plasmids harboring antibiotic resistance genes. Isolates were screened against the dereplication strains using the wagon-wheel assay, in which dereplication strains were streaked in a circle to form the “wheel” on LB plates, and Arctic isolates were streaked across the wheel to form “spokes” (Figure 2B). Plates were incubated for 1 week at room temperature and it was subsequently observed in which cases the isolates were able to inhibit the growth of the dereplication strain. Isolates able to inhibit the growth of all dereplication strains were considered to have “passed.”

### Screening Arctic Isolates That Passed the ARP Against ESKAPE Pathogens

Arctic isolates that passed the ARP were screened for inhibitory activities against a panel of ESKAPE pathogens, namely *Enterobacter cloacae* (strain CO794 provided by the G. Wright laboratory), methicillin-resistant *S. aureus* (MRSA) (ATCC 43300), methicillin-susceptible *S. aureus* (MSSA) (ATCC 29213), *K. pneumoniae* (clinical strain HO132323 provided by the G. Wright laboratory)*, Acinetobacter baumanii* AB5075*, P. aeruginosa* WT PAO1*, E. faecium* (ATCC 700221) and *Enterococcus faecalis* (ATCC 29212), using the wagon-wheel co-culture technique as described above (Figure 2B). Frozen glycerol stock cultures of the Arctic isolates and of the tester pathogens were streaked on LB plates. The pathogen tester strains were incubated at 37°C for 18 h whereas Arctic isolates were incubated at room temperature for 48 h. Once sufficient growth appeared on plates, liquid cultures of the Arctic isolates and the pathogenic tester strains were made using LB media. Liquid cultures of the Arctic isolates were incubated with agitation at 250 rpm for 48 h at room temperature whereas pathogenic tester strains were incubated with agitation at 250 rpm for 24 h at 37°C. Loopfuls of the pathogenic strains in liquid culture were streaked in a circular line on a LB plate, while the Arctic isolates were streaked outward starting from the middle of the plate (Figure 2B). Triplicates of these co-culture plates were incubated at room temperature, and observed after 24, 48, and 72 h of incubation. Regions of contact between the Arctic isolate and pathogenic tester strain in which the tester strain did not produce visible growth were recorded as inhibition.

### Assessing Growth of Arctic Isolates That Passed the ARP at Various Temperatures

Liquid TSB cultures of Arctic isolates were incubated with agitation at 250 rpm for 48 h at room temperature and then streaked on TSA plates. Inoculated plates were then incubated at 37, 25, 10, 5, 0, –5, and –10°C. TSA media intended for sub-zero incubation was amended with 3% v/v glycerol to avoid complete freezing of the media. Growth was visually assessed after 48 h of incubation at 37 and 25°C. The remaining incubations were assessed for growth after 20 days.

### Screening Arctic Isolates That Passed the ARP Against Foodborne Pathogens

In addition to screening against clinical pathogens, all Arctic isolates that passed the dereplication assay were screened against foodborne pathogens using an overlay co-culture assay (Figure 2C). Liquid TSB cultures of Arctic isolates were incubated with agitation at 250 rpm for 2 days at room temperature and then streaked in a line in the center of the TSA plates. The TSA plates were then left to incubate at room temperature for 7 days. Following incubation, each Arctic isolate was overlaid with 7 ml of molten agar media inoculated with 100 μl of overnight liquid cultures of *S. aureus* WT, *Listeria monocytogenes* HPB# 1870 serotype 1/2c, *Salmonella enterica* serovar Heidelberg, and *E. coli* 0157:H7. To assess whether any of the secreted secondary metabolites produced by the Arctic isolates retained inhibitory activity at human body and standard refrigeration temperatures, the overlay plates were incubated at 37 and 5°C, respectively. Zones of inhibition were observed after 24 h of incubation for plates incubated at 37°C and after 20 days for those incubated at 5°C. This assay inherently relies on the diffusion of secreted molecules within the cultivation media’s semisolid agar matrix. Specifically, in order to observe zones of clearance, diffusible biomolecules are required to reach the top agar layer to inhibit the tester strains. This co-culture assay was conducted in triplicate and an established antibiotic-producing strain, *Paenibacillus terrae* NRRL B-30644, was used a positive control. This environmental strain has been previously reported to produce zones of inhibition in co-culture assays against foodborne pathogens (van Belkum et al., 2015). The zones of inhibition produced by *P. terrae* NRRL B-30644 served as a visual reference for positive inhibitory activity for all the co-culture assays conducted in this study (Figure 2C).

### DNA Sequencing, Genome Assembly, and Analyses of Isolates Passing the ARP

Bacterial isolates were inoculated from single isolated colonies into R2A broth and grown at room temperature for periods ranging from 12 to 72 h. The genomic DNA was extracted using the E-Z 96 Tissue DNA Kit (Omega Bio-Tek, Norcross GA, United States). Between 250 and 700 ng of DNA were fragmented via sonication using a Covaris M220 (Covaris, Woburn, MA, United States) for 40 s at 18–22°C. The peak power was set at 50.0, duty factor at 20.0 and the number of bursts at 200. The libraries were prepared using the NEBNext Ultra II DNA library prep kit for Illumina (New England Biolabs, Ipswich MA, United States) following the manufacturer’s instructions. The libraries were barcoded with TruSeq HT adapters (Illumina, San Diego, CA, United States) and sequenced using an Illumina MiSeq 300 bp paired-end run at the Plateforme d’Analyses Génomiques at the Institut de Biologie Intégrative et des Systèmes (Université Laval, Québec, Canada). The raw reads were assembled using the A5 pipeline (Tritt et al., 2012). Assemblies were validated using the metagenomic identification software Centrifuge and contig uniformity was verified to confirm that each assembly originated from the same source DNA and from not contaminants (Kim et al., 2016). To taxonomically identify the isolates that inhibited the ARP, their assembled genomes were first annotated using the Rapid Annotation using Subsystem Technology (RAST) version 2.0 using standard parameters (Aziz et al., 2008; Overbeek et al., 2013; Brettin et al., 2015). Following annotation, full 16S rRNA gene(s) were identified within each genome and subsequently used as a query sequences in the EzBioCloud database for corroborating taxonomical identification (Yoon et al., 2017). To predict the secondary metabolite BGCs within the genomes of the eight isolates of interest, all genomes were mined using the antiSMASH database v.4 using default search parameter settings (Medema et al., 2011; Blin et al., 2017). The BGCs of the isolates showing the broadest activity spectra were used as query sequence in a blastn alignment against genomes of type strains of the same species: *Pseudomonas prosekii* LMG 26867 (genome accession GCA_900105155.1), and *P. terrae* NRRL B-30644 (accession GCA_000943545.1). The assembled genomes of these two promising isolates were deposited at GenBank under the BioSample accessions (SAMN12211638 and SAMN12211639).

### Organic Extraction Using Liquid Culture Supernatants From Two Promising Isolates

To confirm whether the antagonistic activities of promising isolates were caused by secreted secondary metabolites of bacterial origin, organic extracts using liquid culture supernatants were prepared. Isolates were incubated overnight in TSB media at room temperature with agitation at 250 rpm. Following overnight incubation, 14 mL of the cultures were mixed with 7.5 mL of ethyl acetate in a 50 mL Falcon tube (giving a final volume ratio of 2:1 culture supernatant to ethyl acetate). Solutions were vortexed on the highest setting until solutions were homogenized and then centrifuged at 7830 rpm for 15 min at room temperature. Following centrifugation, the organic layers were removed from both solutions and were collected in separate pre-weighed 7.5 mL borosilicate glass culture tubes using a Pasteur pipette. The ethyl acetate was then left to evaporate for 20 min by direct air filtered with a 0.3 μm HEPA filter (Whatman #0974479). After the solvent fully evaporated, both extracts were resuspended in 200 μL of methanol. To assess whether the crude organic extracts retained antibacterial activity, 30 μL of the extracts were spotted on agar plates. Once the methanol fully evaporated from the agar, 7 ml of molten agar containing the tester strains (*E. coli* ATCC 11775 and *E. coli* Δ*bam*Δ*tolC* BW25113) were poured over the dried extract spots. Control spots consisting only of methanol were plated to confirm that the observed zones of clearance did not arise from the solvent used to resuspend the extract. The plates were then incubated at room temperature for 24 h before being observed for zones of clearance.

## Results

### Antibiotic Activity of Arctic Isolates Against ESKAPE Pathogen Relatives

Twelve environmental samples were collected in the regions of Expedition Fiord, Gypsum Hill, Lost Hammer, Color Lake and White Glacier on Axel Heiberg Island in the Canadian high Arctic. The sampled habitats included active layer permafrost, sediments, and lithic communities. Active layer permafrost samples included trough soil from polygonal tundra terrain, surface soil from polygonal tundra terrain, arid soil, and hummock soil. Sediment samples included perennial hypersaline spring outflow channel sediment, acidic freshwater lake sediment, acidic freshwater lake sediment covered with microbial mats, perennial saline spring sediment, and sediment from glacial terminus moraines. Lithic communities included gypsum cryptoendoliths and unknown rock cryptoendoliths (Table 1). All of these samples were distributed to the undergraduate students for traditional plate cultivation, and 3120 distinct colonies were subcultured and further tested. After co-culture screening, 54 isolates (1.7%) were identified as having antibacterial activity against at least one ESKAPE pathogen relative. Of these, three isolates originated from acidic freshwater lake sediment covered with microbial mat, four from a gypsum cryptoendolith, seven from Gypsum Hill hummock active layer permafrost, three from Gypsum Hill perennial saline spring sediment, one from a cryptoendolith collected at Lost Hammer, 25 from active layer tundra permafrost, seven from arid active layer permafrost, two from polygonal terrain surface soil, one from polygonal terrain trough soil, and one from glacial terminus moraine sediment (Table 2). The genera represented by these 54 isolates were *Pseudomonas* (27 isolates), *Bacillus* (5), *Nocardia* (6), *Janthinobacterium* (3), *Streptomyces* (3), *Paenibacillus* (2), *Mycetocola* (1), *Flavobacterium* (1), *Micrococcus* (1), *Curtobacterium* (1), *Arthrobacter* (1), *Pseudoarthrobacter* (1), *Rahnella* (1), and *Siccibacter* (1).

**TABLE 2:** Antibiotic activity of bulk soil isolates against ESKAPE pathogen relatives.

**Source location**	**Habitat**	**Isolate name**	**Closest matching taxon**	**%identity**	**%coverage of 16S gene**	***S*. *epidermidis***	***E*. *raffinosus***	***E*. *coli***	***P*. *putida***	***A*. *baylyi***	***E*. *aerogenes***
Color Lake,	Acidic freshwater	CLMS.5.EMGM.4	*Janthinobacterium lividum*	99.87	54.6	rgb] 1, 0, 0	rgb] 0, 0, 0	rgb] 0, 0, 0	rgb] 0, 0, 0	rgb] 0, 0, 0	rgb] 0, 0, 0
Expedition Fiord	lake sediment	CLMS.5.EMGM.9	*Pseudomonas yamanorum*	100	54.9	rgb] 0, 0, 0	rgb] 0, 0, 0	rgb] 0, 0, 0	rgb] 1, 0, 0	rgb] 0, 0, 0	rgb] 0, 0, 0
	covered with	CLMS.6.VMCS.4	*Janthinobacterium lividum*	99.87	29.6	rgb] 0, 0, 0	rgb] 0, 0, 0	rgb] 0, 0, 0	rgb] 1, 0, 0	rgb] 0, 0, 0	rgb] 0, 0, 0
	microbial mat										
Gypsum Hill,	Gypsum	GHCE.C1.AWCA.27	*Streptomyces avidinii*	100	48.2	rgb] 1, 0, 0	rgb] 0, 0, 0	rgb] 0, 0, 0	rgb] 0, 0, 0	rgb] 0, 0, 0	rgb] 0, 0, 0
Expedition Fiord	cryptoendolith	GHCE.C1.AWCA.31	*Mycetocola miduiensis*	99.75	56.3	rgb] 1, 0, 0	rgb] 0, 0, 0	rgb] 0, 0, 0	rgb] 0, 0, 0	rgb] 0, 0, 0	rgb] 0, 0, 0
		GHCE.5.JVZL.12	*Pseudomonas fulva*	100	100	rgb] 1, 0, 0	rgb] 1, 0, 0	rgb] 1, 0, 0	rgb] 0, 0, 0	rgb] 1, 0, 0	rgb] 0, 0, 0
		GHCE.5.JVZL.15	*Streptomyces avidinii*	100	46.4	rgb] 1, 0, 0	rgb] 0, 0, 0	rgb] 1, 0, 0	rgb] 0, 0, 0	rgb] 1, 0, 0	rgb] 0, 0, 0
	Hummock soil	GHHS.3.LBZX.4	*Flavobacterium panaciterrae*	99	100	rgb] 0, 0, 0	rgb] 0, 0, 0	rgb] 1, 0, 0	rgb] 0, 0, 0	rgb] 0, 0, 0	rgb] 0, 0, 0
		GHHS.3.LBZX.18	*Janthinobacterium svalbardensis*	100	59.5	rgb] 0, 0, 0	rgb] 0, 0, 0	rgb] 1, 0, 0	rgb] 0, 0, 0	rgb] 0, 0, 0	rgb] 0, 0, 0
		GHHS.LB.ZXLB.24	*Pseudomonas helmanticensis*	99.75	54.8	rgb] 0, 0, 0	rgb] 0, 0, 0	rgb] 0, 0, 0	rgb] 1, 0, 0	rgb] 0, 0, 0	rgb] 0, 0, 0
		GHHS.LB.ZXLB.15	*Pseudomonas helmanticensis*	100	54	rgb] 0, 0, 0	rgb] 0, 0, 0	rgb] 0, 0, 0	rgb] 1, 0, 0	rgb] 0, 0, 0	rgb] 0, 0, 0
		GHHS.LB.ZXLB.15	*Pseudomonas arsenicoxydans*	100	54.8	rgb] 0, 0, 0	rgb] 0, 0, 0	rgb] 0, 0, 0	rgb] 1, 0, 0	rgb] 0, 0, 0	rgb] 0, 0, 0
		GHHS.LB.ZXLB.9	*Pseudomonas arsenicoxydans*	100	46.2	rgb] 0, 0, 0	rgb] 0, 0, 0	rgb] 0, 0, 0	rgb] 1, 0, 0	rgb] 0, 0, 0	rgb] 0, 0, 0
		GHHS.13.PTLY.2	*Pseudomonas arsenicoxydans*	100	53.9	rgb] 1, 0, 0	rgb] 0, 0, 0	rgb] 0, 0, 0	rgb] 0, 0, 0	rgb] 0, 0, 0	rgb] 0, 0, 0
	Perennial saline	GHS.C1.RZCG.3	*Bacillus nakamurai*	100	45.9	rgb] 1, 0, 0	rgb] 0, 0, 0	rgb] 0, 0, 0	rgb] 0, 0, 0	rgb] 0, 0, 0	rgb] 0, 0, 0
	spring sediment	GHS.C1.RZCG.16	*Micrococcus aloeverae*	99.85	46.8	rgb] 1, 0, 0	rgb] 0, 0, 0	rgb] 0, 0, 0	rgb] 0, 0, 0	rgb] 0, 0, 0	rgb] 0, 0, 0
		GHS.8.NWYW.5	*Paenibacillus terrae*	99.23	100	rgb] 0, 0, 0	rgb] 0, 0, 0	rgb] 1, 0, 0	rgb] 0, 0, 0	rgb] 0, 0, 0	rgb] 0, 0, 0
Junction Diapir (Lost Hammer)	Unknown rock cryptoendolith	LCE.5.TZJR.21	*Pseudomonas lurida*	99.87	54.7	rgb] 1, 0, 0	rgb] 0, 0, 0	rgb] 0, 0, 0	rgb] 0, 0, 0	rgb] 0, 0, 0	rgb] 0, 0, 0
MARS station	Active layer	MAL.2.HSSH.5	*Pseudomonas kilonensis*	99.86	48.5	rgb] 1, 0, 0	rgb] 0, 0, 0	rgb] 0, 0, 0	rgb] 0, 0, 0	rgb] 0, 0, 0	rgb] 0, 0, 0
Expedition Fiord	tundra permafrost	MAL.4.ABES.12	*Pseudomonas arsenicoxydans*	99.86	49.6	rgb] 0, 0, 0	rgb] 0, 0, 0	rgb] 1, 0, 0	rgb] 0, 0, 0	rgb] 0, 0, 0	rgb] 0, 0, 0
		MAL.4.ABES.21	*Pseudomonas frederiksbergensis*	100	100	rgb] 0, 0, 0	rgb] 0, 0, 0	rgb] 1, 0, 0	rgb] 0, 0, 0	rgb] 0, 0, 0	rgb] 0, 0, 0
		MAL.11.AHCHQX.12	*Pseudomonas arsenicoxydans*	100	100	rgb] 0, 0, 0	rgb] 0, 0, 0	rgb] 1, 0, 0	rgb] 0, 0, 0	rgb] 0, 0, 0	rgb] 0, 0, 0
		MAL.10.WYTK.25	*Pseudomonas extremaustralis*	99.73	100	rgb] 0, 0, 0	rgb] 1, 0, 0	rgb] 1, 0, 0	rgb] 0, 0, 0	rgb] 0, 0, 0	rgb] 0, 0, 0
		C2C25	*Curtobacterium pusillum*	99.54	100	rgb] 0, 0, 0	rgb] 1, 0, 0	rgb] 0, 0, 0	rgb] 0, 0, 0	rgb] 0, 0, 0	rgb] 0, 0, 0
		C5C25	*Pseudomonas migulae*	99.6	100	rgb] 0, 0, 0	rgb] 1, 0, 0	rgb] 1, 0, 0	rgb] 1, 0, 0	rgb] 1, 0, 0	rgb] 1, 0, 0
		HTAG2	*Pseudomonas jessenii*	99.34	100	rgb] 1, 0, 0	rgb] 1, 0, 0	rgb] 0, 0, 0	rgb] 0, 0, 0	rgb] 0, 0, 0	rgb] 0, 0, 0
		C6A2	*Nocardia coeliaca*	99.62	100	rgb] 1, 0, 0	rgb] 0, 0, 0	rgb] 0, 0, 0	rgb] 0, 0, 0	rgb] 0, 0, 0	rgb] 0, 0, 0
		C6A4	*Streptomyces netropsis*	100	100	rgb] 1, 0, 0	rgb] 0, 0, 0	rgb] 0, 0, 0	rgb] 0, 0, 0	rgb] 0, 0, 0	rgb] 0, 0, 0
		C7A1	*Nocardia coeliaca*	99.61	100	rgb] 1, 0, 0	rgb] 0, 0, 0	rgb] 0, 0, 0	rgb] 0, 0, 0	rgb] 0, 0, 0	rgb] 0, 0, 0
		C4F16	*Arthrobacter oryzae*	99.46	100	rgb] 0, 0, 0	rgb] 0, 0, 0	rgb] 1, 0, 0	rgb] 0, 0, 0	rgb] 0, 0, 0	rgb] 0, 0, 0
		C6D6	*Nocardia globerula*	99.34	100	rgb] 0, 0, 0	rgb] 1, 0, 0	rgb] 1, 0, 0	rgb] 0, 0, 0	rgb] 0, 0, 0	rgb] 0, 0, 0
		C6D9	*Pseudoarthrobacter phenanthrenivorans*	99.09	100	rgb] 0, 0, 0	rgb] 1, 0, 0	rgb] 1, 0, 0	rgb] 1, 0, 0	rgb] 0, 0, 0	rgb] 1, 0, 0
		C7B19	*Nocardia coeliaca*	99.61	100	rgb] 0, 0, 0	rgb] 0, 0, 0	rgb] 1, 0, 0	rgb] 1, 0, 0	rgb] 0, 0, 0	rgb] 1, 0, 0
		C7B21	*Bacillus mycoide*	99.69	100	rgb] 0, 0, 0	rgb] 0, 0, 0	rgb] 0, 0, 0	rgb] 1, 0, 0	rgb] 0, 0, 0	rgb] 0, 0, 0
		C7D7	*Paenibacillus xylanexedens*	100	100	rgb] 0, 0, 0	rgb] 0, 0, 0	rgb] 0, 0, 0	rgb] 1, 0, 0	rgb] 0, 0, 0	rgb] 0, 0, 0
		C8B11	*Rahnella inusitata*	98.59	100	rgb] 0, 0, 0	rgb] 0, 0, 0	rgb] 0, 0, 0	rgb] 1, 0, 0	rgb] 0, 0, 0	rgb] 0, 0, 0
		C8C11.16	*Bacillus subtilis*	99.74	100	rgb] 0, 0, 0	rgb] 0, 0, 0	rgb] 1, 0, 0	rgb] 0, 0, 0	rgb] 0, 0, 0	rgb] 0, 0, 0
		C10F22	*Nocardia coeliaca*	99.59	100	rgb] 0, 0, 0	rgb] 0, 0, 0	rgb] 1, 0, 0	rgb] 1, 0, 0	rgb] 0, 0, 0	rgb] 0, 0, 0
		C10F26	*Pseudomonas fluorescens*	99.28	100	rgb] 0, 0, 0	rgb] 0, 0, 0	rgb] 0, 0, 0	rgb] 1, 0, 0	rgb] 1, 0, 0	rgb] 0, 0, 0
		C10G6	*Nocardia globerula*	99.34	100	rgb] 0, 0, 0	rgb] 0, 0, 0	rgb] 0, 0, 0	rgb] 1, 0, 0	rgb] 1, 0, 0	rgb] 0, 0, 0
		C11E10	*Bacillus subtilis*	100	100	rgb] 0, 0, 0	rgb] 0, 0, 0	rgb] 1, 0, 0	rgb] 0, 0, 0	rgb] 0, 0, 0	rgb] 0, 0, 0
		C11E23	*Bacillus tequilensis*	99.87	100	rgb] 0, 0, 0	rgb] 0, 0, 0	rgb] 1, 0, 0	rgb] 0, 0, 0	rgb] 0, 0, 0	rgb] 0, 0, 0
		MAL.11.SPBF.1	*Pseudomonas arsenicoxydans*	99.75	55.9	rgb] 0, 0, 0	rgb] 1, 0, 0	rgb] 0, 0, 0	rgb] 0, 0, 0	rgb] 0, 0, 0	rgb] 0, 0, 0
	Arid soil	AALPS.3.MFAL.10	*Pseudomonas lutea*	100	36	rgb] 0, 0, 0	rgb] 0, 0, 0	rgb] 1, 0, 0	rgb] 0, 0, 0	rgb] 0, 0, 0	rgb] 0, 0, 0
		AALPS.2.EKRD.20	*Pseudomonas mandelii*	99.73	100	rgb] 0, 0, 0	rgb] 0, 0, 0	rgb] 1, 0, 0	rgb] 0, 0, 0	rgb] 0, 0, 0	rgb] 0, 0, 0
		AALPS.4.MSMB.5	*Pseudomonas mandelii*	99.66	100	rgb] 0, 0, 0	rgb] 0, 0, 0	rgb] 1, 0, 0	rgb] 0, 0, 0	rgb] 0, 0, 0	rgb] 0, 0, 0
		AALPS.10.JKNJ.7	*Pseudomonas prosekii*	100	100	rgb] 0, 0, 0	rgb] 0, 0, 0	rgb] 1, 0, 0	rgb] 1, 0, 0	rgb] 0, 0, 0	rgb] 0, 0, 0
		AALPS.10.EMMH.23	*Pseudomonas yamanorum*	98.68	100	rgb] 0, 0, 0	rgb] 0, 0, 0	rgb] 1, 0, 0	rgb] 0, 0, 0	rgb] 0, 0, 0	rgb] 0, 0, 0
		AALPS.10.MNAAK.13	*Pseudomonas prosekii*	99.93	100	rgb] 0, 0, 0	rgb] 0, 0, 0	rgb] 1, 0, 0	rgb] 0, 0, 0	rgb] 0, 0, 0	rgb] 0, 0, 0
		AALPS.13.YAYA.22	*Pseudomonas prosekii*	100	21	rgb] 1, 0, 0	rgb] 0, 0, 0	rgb] 0, 0, 0	rgb] 0, 0, 0	rgb] 0, 0, 0	rgb] 0, 0, 0
	Surface soil from	PSALP.2.BBCP.18	*Pseudomonas lurida*	99.84	42.6	rgb] 0, 0, 0	rgb] 0, 0, 0	rgb] 0, 0, 0	rgb] 0, 0, 0	rgb] 1, 0, 0	rgb] 0, 0, 0
	polygonal tundra	PSALP.2.JQGR.13	*Pseudomonas kilonensis*	99.87	53.8	rgb] 0, 0, 0	rgb] 0, 0, 0	rgb] 0, 0, 0	rgb] 0, 0, 0	rgb] 1, 0, 0	rgb] 0, 0, 0
	terrain										
	Trough soil from polygonal tundra terrain	PWALP.2.KADK.9	*Pseudomonas fluorescens*	100	46.9	rgb] 0, 0, 0	rgb] 0, 0, 0	rgb] 1, 0, 0	rgb] 1, 0, 0	rgb] 0, 0, 0	rgb] 0, 0, 0
White Glacier, Expedition Fiord	Sediment from glacial terminus moraines	GS.C1.SPSB.3	*Siccibacter turicensis*	100	53.9	rgb] 0, 0, 0	rgb] 1, 0, 0	rgb] 0, 0, 0	rgb] 0, 0, 0	rgb] 0, 0, 0	rgb] 0, 0, 0

*Red boxes: inhibitory activity. Black boxes: no inhibitory activity.*

Microbial cultivation using the cryo-iPlate incubated in active layer permafrost soil within the vicinity of the MARS lead to the isolation of ∼300 morphologically distinct colonies. A total of 16 isolates (∼5%) exhibited antibacterial activity against at least one ESKAPE pathogen relative. All cryo-iPlate strains with antibacterial activity only displayed zones of inhibition against *P. putida* except for *Pedobacter* isolate B7.1 that inhibited *S. epidermidis*, and *Flavobacterium* strain C9.2 that inhibited the growth of *E. coli* and *P. putida* (Table 3). Genera represented by these 16 isolates were *Pseudomonas* (4 isolates), *Pedobacter* (3), *Flavobacterium* (3), *Janthinobacterium* (2), *Agreia* (1), *Pararhizobium* (1), *Sphingomonas* (1), and *Stenotrophomonas* (1) (Table 3). The genera isolated by both the classical soil plating approach and the cryo-iPlate were *Pseudomonas, Flavobacterium* and *Janthinobacterium*. Among both bulk plated and cryo-iPlate isolates, *Pseudomonas* represented the genus with the highest proportion of total isolated strains (50% of total bulk plated isolates and 25% of total cryo-iPlate isolates).

**TABLE 3:** Antibiotic activity of cryo-iPlate isolates against ESKAPE pathogen relatives.

**Isolate name**	**Closest match**	**% identity**	**S. e*pidermidis***	**E. *raffinosus***	**E. c*oli***	**P. *putida***	**A. *baylyi***	**E. *aerogenes***
A4.3Y	*Janthinobacterium lividum*	99.31	rgb] 0, 0, 0	rgb] 0, 0, 0	rgb] 0, 0, 0	rgb] 1, 0, 0	rgb] 0, 0, 0	rgb] 0, 0, 0
B1.1W	*Pseudomonas trivialis*	100	rgb] 0, 0, 0	rgb] 0, 0, 0	rgb] 0, 0, 0	rgb] 1, 0, 0	rgb] 0, 0, 0	rgb] 0, 0, 0
B4.6	*Pseudomonas migulae*	100	rgb] 0, 0, 0	rgb] 0, 0, 0	rgb] 0, 0, 0	rgb] 1, 0, 0	rgb] 0, 0, 0	rgb] 0, 0, 0
B7.1	*Pedobacter humicola*	99.01	rgb] 1, 0, 0	rgb] 0, 0, 0	rgb] 0, 0, 0	rgb] 0, 0, 0	rgb] 0, 0, 0	rgb] 0, 0, 0
C3.3	*Pedobacter alluvionis*	99.09	rgb] 0, 0, 0	rgb] 0, 0, 0	rgb] 0, 0, 0	rgb] 1, 0, 0	rgb] 0, 0, 0	rgb] 0, 0, 0
C6.2	*Pseudomonas koreensis*	100	rgb] 0, 0, 0	rgb] 0, 0, 0	rgb] 0, 0, 0	rgb] 1, 0, 0	rgb] 0, 0, 0	rgb] 0, 0, 0
C7.3	*Agreia pratensis*	100	rgb] 0, 0, 0	rgb] 0, 0, 0	rgb] 0, 0, 0	rgb] 1, 0, 0	rgb] 0, 0, 0	rgb] 0, 0, 0
D4.1	*Pseudomonas arsenicoxydans*	100	rgb] 0, 0, 0	rgb] 0, 0, 0	rgb] 0, 0, 0	rgb] 1, 0, 0	rgb] 0, 0, 0	rgb] 0, 0, 0
G2.2	*Flavobacterium oncorhynchi*	100	rgb] 0, 0, 0	rgb] 0, 0, 0	rgb] 0, 0, 0	rgb] 1, 0, 0	rgb] 0, 0, 0	rgb] 0, 0, 0
F9.3	*Pedobacter terrae*	95.59	rgb] 0, 0, 0	rgb] 0, 0, 0	rgb] 0, 0, 0	rgb] 1, 0, 0	rgb] 0, 0, 0	rgb] 0, 0, 0
D1.3	*Janthinobacterium lividum*	99.25	rgb] 0, 0, 0	rgb] 0, 0, 0	rgb] 0, 0, 0	rgb] 1, 0, 0	rgb] 0, 0, 0	rgb] 0, 0, 0
C12.2A	*Stenotrophomonas rhizophila*	99.49	rgb] 0, 0, 0	rgb] 0, 0, 0	rgb] 0, 0, 0	rgb] 1, 0, 0	rgb] 0, 0, 0	rgb] 0, 0, 0
C12.1B	*Flavobacterium frigidimaris*	99.2	rgb] 0, 0, 0	rgb] 0, 0, 0	rgb] 0, 0, 0	rgb] 1, 0, 0	rgb] 0, 0, 0	rgb] 0, 0, 0
C11.2	*Pararhizobium herbae*	100	rgb] 0, 0, 0	rgb] 0, 0, 0	rgb] 0, 0, 0	rgb] 1, 0, 0	rgb] 0, 0, 0	rgb] 0, 0, 0
C11.3	*Sphingomonas aerolata*	99.68	rgb] 0, 0, 0	rgb] 0, 0, 0	rgb] 0, 0, 0	rgb] 1, 0, 0	rgb] 0, 0, 0	rgb] 0, 0, 0
C9.2	*Flavobacterium hydatis*	100	rgb] 0, 0, 0	rgb] 0, 0, 0	rgb] 1, 0, 0	rgb] 1, 0, 0	rgb] 0, 0, 0	rgb] 0, 0, 0

*Red boxes: positive inhibitory activity. Black boxes: negative inhibitory activity.*

**TABLE 4:** Identification of secondary metabolite biosynthetic gene clusters within genomes of isolates inhibiting the ARP.

**Isolate**	**Genome size (Mb)**	**Isolate Identification**	**%16S gene ID**	**AntiSMASH (total clusters)**	**NRP^1^**	**PK^2^**	**Bacterion/RiPP^3^**	**Trans PKS^4^**	**Other**
GHHS.3.LBZX.4	6.1	*Flavobacterium panaciterrae*	87.54	6	1	1	0	0	4
GHS.8.NWYW.5	5.7	*Paenibacillus terrae*	99.23	23	17	0	3	1	2
MAL.10.WYTK.25	6.8	*Pseudomonas extremaustralis*	99.73	9	4	0	2	0	3
GHCE.5.JVZL.12	5	*Pseudomonas fulva*	100	11	9	0	0	0	2
AALPS.4.MSMB.5	6.3	*Pseudomonas mandelii*	99.66	10	3	0	3	1	3
AALPS.10.MNAAK.13	6.1	*Pseudomonas prosekii*	99.93	8	2	0	1	0	5
MAL.4.ABES.21	7.1	*Pseudomonas frederiksbergensis*	99.86	7	3	0	1	0	3
C11E23	4.3	*Bacillus tequilensis*	100	12	4	1	3	1	3

*^1^Number of non-ribosomal peptide gene clusters identified using AntiSMASH. ^2^Number of polyketide gene clusters identified using AntiSMASH. ^3^Number of bacteriocin and ribosomally synthesized and post-translationally modified peptides (RiPP) identified using AntiSMASH. ^4^Number of trans-polyketide gene clusters identified using AntiSMASH.*

### Antibacterial Activity of Arctic Isolates That Passed the ARP Against Clinical Pathogens

Eight (8) out of the total seventy (70) antibiotic-producing isolates (11%) were found to inhibit the growth of all strains comprising the ARP. These strains were prioritized for further investigation. To assess whether these isolates were capable of inhibiting the growth of clinically significant pathogens, the isolates were screened against ESKAPE organisms *P. aeruginosa*, MRSA, *A. baumanii*, MSSA, *E. cloacae*, *K. pneumoniae*, *E. faecium*, and *E. faecalis*. *Pseudomonas* sp. AALPS.10.MNAAK.13 was observed to have the broadest antibacterial activity spectrum. It inhibited the growth of MRSA, *A. baumanii*, MSSA, *E faecium*, and *E. faecalis* (Table 5). *Flavobacterium* sp. GHHS.3.LBZX.4 and *Pseudomonas* sp. MAL.4.ABES.21 were not observed to inhibited any of the pathogenic tester strains. *Pseudomonas* sp. MAL.10.WYTK.25, *Pseudomonas* sp. AALPS.4.MSMB.5 and *Bacillus* sp. C11E23 inhibited the growth of MRSA, MSSA, and *E. faecium*. *Paenibacillus* sp. GHS.8.NWYW.5 was capable of inhibiting MRSA and MSSA. Pseudomonas sp. GHCE.5JVZL.12 inhibited the growth of MRSA, *A. baumanii*, MSSA, and *K. pneumoniae*.

**TABLE 5:** Screening Arctic isolates that inhibited the ARP against clinical pathogens.

**Isolate name**	**Closest matching species**	**Closest matching strain**	***P. aeruginosa***	***MRSA***	***A. baumanii***	***MSSA***	***E. cloacae***	***K. pneumoniae***	***E. faecium***	***E. faecalis***
GHHS.3.LBZX.4	*Flavobacterium panaciterrae*	DCY69(T)								
GHS.8.NWYW.5	*Paenibacillus terrae*	AM141(T)		+		+				
AALPS.10.MNAAK.13	*Pseudomonas prosekii*	LMG 26867		+	+	+			+	+
MAL.10.WYTK.25	*Pseudomonas extremaustralis*	14-3(T)		+		+			+	
GHCE.5.JVZL.12	*Pseudomonas fluorescens*	DSM 50090(T)		+	+	+		+		
AALPS.4.MSMB.5	*Pseudomonas mandelii*	NBRC 103147(T)		+		+			+	
MAL.4.ABES.21	*Pseudomonas frederikbergensis*	JAJ28(T)								
C11E23	*Bacillus tequilensis*	KCTC 13622(T)		+		+			+	

*(+) Positive inhibitory activity.*

### Growth of Arctic Isolates That Passed the ARP at Various Temperatures

After screening against clinically significant pathogens, we assessed whether the eight prioritized isolates were capable of growing at various temperatures (37, 25, 10, 5, 0, and –5°C). All eight isolates were capable of growth at 5°C. Only two isolates (*Paenibacillus* sp. GHS.8.NWYW.5 and *Pseudomonas* sp. GHCE.5.JVZL.12) were capable of growing at 37°C after 48 h of incubation. All isolates except *Pseudomonas* sp. GHCE.5.JVZL.12 grew at 0°C after 20 days of incubation. With the exception of *Flavobacterium* sp. GHHS.3.LBZX.4 and *Pseudomonas* sp. GHCE.5.JVZL.12, all other Arctic isolates that passed the ARP were capable of growing at –5°C after 20 days of incubation (Supplementary Table S3).

### Antibacterial Activity of Prioritized Isolates Against Foodborne Pathogens

The eight prioritized isolates were tested for antibacterial activity against four foodborne pathogens (*S. aureus*, *L. monocytogenes*, *S. enterica* and *E. coli* O157:H7 at two different incubation temperatures (37 and 5°C). *Paenibacillus* sp. GHS.8.NWYW.5 was observed to inhibit the growth of *S. aureus*, *L. monocytogenes*, *S. enterica*, and *E. coli* O157:H7 at 37 and 5°C. *Pseudomonas* sp. AALPS.10.MNAAK.13 was observed to inhibit *S. aureus* at 37°C after 24 h of incubation. *Pseudomonas* sp. MAL.10.WYTK.25 was observed to inhibit the growth of *S. enterica at* 37°C after 24 h. *Bacillus* sp. C11E23 inhibited *L. monocytogenes* at 37 and 5°C (Table 6). The zones of inhibition produced by these Arctic isolates were consistent with the known antibiotic-producing strain *P. terrae* NRRL B-30644 which displayed inhibitory activity against all foodborne pathogens used in this assay.

**TABLE 6:** Screening Arctic isolates that inhibited the ARP against foodborne pathogens.

			***Staphylococcus***		***Listeria***		***Salmonella***		***Escherichia***	
			***aureus***		***monocytogenes***		***enterica***		***coli O157:H7***	
**Isolate name**	**Closest matching species**	**Closest matching strain**	**37*°*C**	**5°C**	**37*°*C**	**5°C**	**37*°*C**	**5°C**	**37*°*C**	**5°C**
GHHS.3.LBZX.4	*Flavobacterium panaciterrae*	DCY69(T)								
GHS.8.NWYW.5	*Paenibacillus terrae*	AM141(T)	+	+	+	+	+	+	+	+
AALPS.10.MNAAK.13	*Pseudomonas prosekii*	LMG 26867	+							
MAL.10.WYTK.25	*Pseudomonas extremaustralis*	14-3(T)					+			
GHCE.5.JVZL.12	*Pseudomonas fluorescens*	DSM 50090(T)								
AALPS.4.MSMB.5	*Pseudomonas mandelii*	NBRC 103147(T)								
MAL.4.ABES.21	*Pseudomonas frederikbergensis*	JAJ28(T)								
C11E23	*Bacillus tequilensis*	KCTC 13622(T)			+	+				

*(+) Positive inhibitory activity.*

### Genomic Analysis of Bacterial Isolates Capable of Inhibiting the Entire ARP

After the Illumina MiSeq 300 bp paired-end run, the range of number of raw reads was 665502–2929247 and the fragment size range was 400–2000 bp (Supplementary Table S4 and Supplementary Figure S1). The genomes of the eight prioritized isolates were then mined using the antiSMASH database to identify secondary metabolite gene clusters that they encode (Table 4). These genomes contained ≥ 6 putative BGCs that were homologous to NRP, PK, bacteriocin/ribosomally synthesized and post-translationally modified peptide (RiPP), and trans-polyketide synthetase (trans-PKS). All clusters labeled as “other” corresponded to either arylpolyene, terpene, betalactone, siderophore, N-acetylglutaminylglutamine (NAGGN) amide or phosphonate clusters (not shown in table). The genome of *Paenibacillus* sp. GHS.8.NWYW.5 featured the greatest number of BGCs compared to all other isolates with 23 secondary metabolite clusters. These clusters included 17 NRP clusters, three bacteriocin/RiPP clusters, one trans-PKS cluster, and two other clusters (one siderophore and one phosphonate cluster). The isolate featuring the least number of BGCs within its genome was *Flavobacterium* sp. GHHS.3.LBZX.4 with a total of six secondary metabolite clusters. These included one type-3 PK, one NRP and four other clusters (one arylpolyene cluster, two terpene clusters, and one betalactone cluster). *Pseudomonas* sp. AALPS.10.MNAAK.13 and *Paenibacillus* sp. GHS.8.NWYW.5 represent the two isolates that displayed the broadest spectra of antibacterial activities against foodborne and clinical pathogens. Eight BGCs were identified within the genome of *Pseudomonas* sp. AALPS.10.MNAAK.13 using antiSMASH (Table 7). After conducting a blastn alignment between the isolate’s clusters with the *Pseudomonas prosekii* LMG 26867 type strain genome, none of the detected clusters displayed low homology within the type strain genome. The genome of the *Paenibacillus* sp. GHS.8.NWYW.5 featured 23 BGCs (Table 7). The clusters with low homology (<50% coverage, <30% identity, and e-values > 0) to the type strain genome of *P. terrae* NRRL B-30644 included: one NRP cluster displayed 11% coverage and 98% identity, one NRP cluster matching to pelgipeptin displayed 45% coverage and 97% identity, one NRP cluster displayed 41% coverage and 75% identity, and two unknown NRP clusters that did not have any significant homology with the type strain genome.

**TABLE 7:** Comparing secondary metabolite gene clusters identified within genomes of promising isolates with type strains of the same species.

**Isolate^a^**	**Type strain genome^b^**	**BGC category^c^**	**Closest matching known BGC^d^**	**Coverage (%)^e^**	**ID (%)^f^**	**e-value^g^**
AALPS.10.MNAAK.13 (*Pseudomonas prosekii*)	*Pseudomonas prosekii* LMG 26867	NRP	pyoverdine	95	97.99	0
		betalactone	fengycin	100	98.3	0
		terpene	–	66	99.03	0
		arylpolyene	APE vf	95	98.63	0
		hserlactone	–	99	98.87	0
		bacteriocin	bacillomycin	100	98.22	0
		NAGGN	–	100	98.63	0
		NRP	pyoverdine	99	98.40	0
GHS.8.NWYW.5 (*Paenibacillus terrae*)	*Paenibacillus terrae* NRRL B-30644	NRP	tridecaptin	87	91	0
		siderophore	staphylobactin	88	95	0
		lanthipeptide	paenicidin B	100	96	0
		phosphonate	–	58	95	0
		lassopeptide	–	100	95	0
		NRP	–	54	93	0
				40	93	0
		NRP	calicheamicin	89	95	0
		NRP trans-ATPKS	paenilarvin	71	93	0
		NRP-betalactone	–	60	91	0
		NRP	–	58	94	0
		NRP		11	98	0
		bacteriocin	–	93	92	0
		NRP	fusaricidin	99	93	0
				37	65	0
		NRP	pelgipeptin	45	97	0
		NRP	–	*NA*	NA	NA
		NRP	–	41	75	0
		NRP	–	*NA*	NA	NA
		NRP	polymyxin	62	69	0
				44	69	7e-141
		NRP	tridecaptin	55	69	0
		NRP	–	46	73	8e-36
		NRP	–	95	73	0
				65	70	5e-38
				46	67	1e-33
		NRP	paenibacterin	38	74	1e-35
		NRP	–	61	69	3e-38

*^*a*^Prioritized isolates displaying widest spectra of antagonistic activity against foodborne and ESKAPE pathogens. ^*b*^Corresponding type strain genome of isolates’ species. ^*c*^Category of secondary metabolite BGCs within genomes of isolates identified using the antiSMASH database. ^*d*^Closest match to known secondary metabolite clusters within isolates’ genomes. ^*e*^Percent coverage of Arctic isolates’ BGCs within reference genomes. ^*f*^Percent identity of Arctic isolates’ BGCs within reference genomes. ^*g*^e-value of Arctic isolates’ BGCs within reference genomes.*

### Organic Extraction Using Liquid Culture Supernatants

**FIGURE 3:**
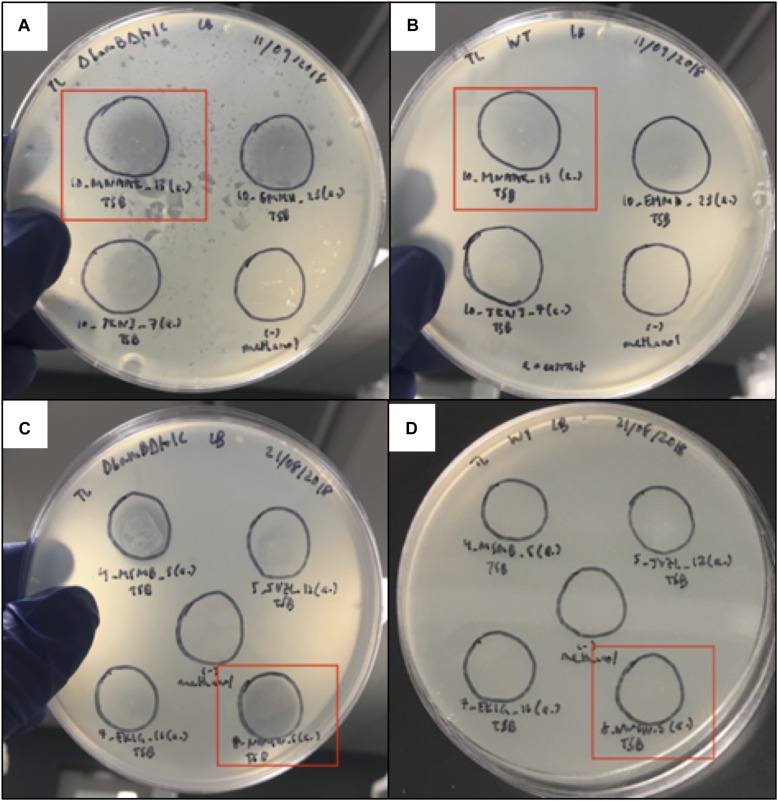
Spot assay used to assess antibacterial activity of organic extracts derived from Arctic isolate supernatants. **(A,B)** Organic extracts of isolates *Pseudomonas* sp. AALPS.10.MNAAK.13, *Pseudomonas* sp. AALPS.10.EMMH.23 and *Pseudomonas* sp. AALPS.10.JKNJ.7 tested against both parental strains of the dereplication platform (*E. coli* Δ*bam*Δ*tolC* BW25113 and *E. coli* BW25113, respectively). Promising isolate *Pseudomonas* sp. AALPS.10.MNAAK.13 that displayed the broadest antibacterial activity in co-culture assays is boxed in red. Negative control spots solely consisting of the methanol are displayed at the bottom right in panels **(A,B)**. **(C,D)** Organic extracts of isolates *Paenibacillus* sp. GHS.8.NWYW.5, *Pseudomonas* sp. AALPS.4.MSMB.5, *Pseudomonas* sp. GHCE.5.JVZL.12, *Pseudomonas* sp. MAL.10.WYTK.25 (same isolate as 7.EKIG.16 in figure) tested against both parental strains of the dereplication platform (*E. coli* Δ*bam*Δ*tolC* BW25113 and *E. coli* BW25113, respectively). Promising isolate *Paenibacillus* sp. GHS.8.NWYW.5 that displayed the broadest antibacterial activity in co-culture assays is boxed in red. Negative control spots solely consisting of methanol are displayed in the center of panels **(C,D)**.

Organic extracts using liquid culture supernatants from isolates *Paenibacillus* sp. GHS.8.NWYW.5, *Pseudomonas* sp. AALPS.10.MNAAK.13, *Pseudomonas* sp. AALPS.10.EMMH.23, *Pseudomonas* sp. AALPS.10.JKNJ.7, *Pseudomonas* sp. AALPS.4.MSMB.5, *Pseudomonas* sp. GHCE.5.JVZL.12, *Pseudomonas* sp. MAL.10.WYTK.25 (same isolate as 7.EKIG.16 in Figure 3) were prepared using ethyl acetate. The crude organic extracts were then tested for retained antibacterial activity using a plate-based spot assay against the parental strains of the ARP (*E. coli* Δ*bam*Δ*tolC* BW25113 and *E. coli* BW25113). After 24 h of incubation at room temperature, the extracts of *Pseudomonas* sp. AALPS.10.MNAAK.13, *Pseudomonas* sp. AALPS.10.EMMH.23 and *Paenibacillus* sp. GHS.8.NWYW.5 exhibited zones of inhibition against *E. coli* Δ*bam*Δ*tolC* BW25113 (Figures 3A,D). The extract of *Pseudomonas* sp. AALPS.10.EMMH.23, yielded a zone of inhibition against *E. coli* BW25113 (Figure 3B).

## Discussion

### Arctic Bacteria Demonstrate Antibiotic Activity Against ESKAPE Pathogen Relatives

Bacteria capable of inhibiting ESKAPE pathogen relatives were isolated from all sampled Arctic environments, with the exception of perennial hypersaline spring sediment sample from Lost Hammer. A total of 70 antibiotic producing isolates were identified, with 54 coming from a classical bulk soil plating approach, and 16 from cryo-iPlate cultivation. More isolates were obtained from the bulk plating approach aided by the greater throughput of approximately 130 undergraduate students processing samples in parallel, while the cryo-iPlate samples were processed by only two graduate students. Additionally, the volume of Arctic samples used for classical plating to isolate microorganisms was ∼60 times greater than that used to inoculate the cryo-iPlate. None of the isolates with antibacterial activity were obtained from perennial hypersaline spring sediment from Lost Hammer, which may be because the salt content of the cultivation media was not adjusted to select for halophilic strains. It is known that the perennial hypersaline spring sediment at Lost Hammer hosts an active microbial community including members of the *Loktanella*, *Gillisia*, *Halomonas* and *Marinobacter* genera (Niederberger et al., 2010), and lack of isolates therefore cannot be attributed to lack of viable bacteria in the source material.

The sample type that yielded the greatest number of isolates with antibiotic activity was active layer soils overlaying permafrost, which consists of a low carbon mineral cryosol. It is ∼60 cm deep during the Arctic summer and completely freezes during winter and spring (Doran, 1993; Lau et al., 2015; Goordial et al., 2017). Previously cultured phylotypes from this habitat predominantly included Actinobacteria (Niederberger et al., 2010), and indeed 26% of isolates identified by the bulk soil plating approach belonged to the Actinobacteria. This phylum includes the genus *Streptomyces* which are known to be prolific producers of antibiotics (Kiesser et al., 2000; Manivasagan et al., 2014). A previous study using functional metagenomics showed that antibiotic resistance genes are present in similar permafrost soils at nearby Eureka, Ellesmere Island (Perron et al., 2015). The presence of antibiotics will create selective pressure for the development of antibiotic resistance. The presence of both antibiotic activity and antibiotic resistance therefore suggests interactions between these functions in the microbial permafrost community.

All cryo-iPlate strains were derived from active layer permafrost soil, because this was the only sample type in which the cryo-iPlate was incubated. The cryo-iPlate yielded genera (*Pararhizobium*, *Sphingomonas*, *Stenotrophomonas*, *Agreia*, *Pedobacter*) that were not observed in the strains isolated by the classical method from the same environment. In the initial implementation of the cryo-iPlate prototype, many of the same genera were isolated, including a putatively novel *Pedobacter* strain (Goordial et al., 2017). Similarly, *Pedobacter* strain F9.3 derived from the cryo-iPlate in this study was found to have 95.6% 16S rRNA gene sequence identity to its closest match *Pedobacter terrae* (Table 3). While none of the cryo-iPlate derived strains inhibited the entire ARP, they were able to inhibit the growth of both Gram-positive and Gram-negative relatives of the ESKAPE pathogens. Given that these strains were isolated from cryohabitats, their antibacterial activity has potential applications in food safety, where inhibitory activity is required at refrigeration temperatures.

### Eight Arctic Bacterial Isolates Inhibited All ARP Strains

Here we have identified eight isolates able to inhibit the ARP which corresponded to either Firmicutes, Bacteroidetes or γ-Proteobacteria. It is worth noting that the dereplication method employed in this study is not a comprehensive approach in identifying isolates expressing novel antibiotic compounds. For example, if a given antibiotic-producing isolate expresses more than one antimicrobial secondary metabolite, it would be capable of inhibiting the growth of all strains comprising the ARP regardless of whether the mechanisms of action of its secondary metabolites are novel. Given this inherent limitation of the ARP, the benefit of using this platform is an initial quick and cost-effective method of screening isolates with inhibitory activities for potentially novel activities. Further identification and characterization of the causative agents responsible for the inhibitory activities are required to confirm the novelty of a natural product.

The phyla of the prioritized isolates are known to make up a significant proportion of the active layer permafrost microbial community and are established secondary metabolite producers (Pathma et al., 2011; Wilhelm et al., 2011). Five out of the eight isolates that passed dereplication were *Pseudomonas* spp. This observation is not unexpected, as these Gram-negative heterotrophic γ-Proteobacteria are routinely isolated from soil samples using classical cultivation techniques such as the ones employed here (Sbrana et al., 2002). Additionally, the microbial community living in active layer permafrost within the vicinity of the MARS has been found to contain 19.4% γ-Proteobacteria (Wilhelm et al., 2011).

The only isolate that passed dereplication pertaining to the phylum Bacteroidetes was *Flavobacterium* sp. GHHS.3.LBZX.4. Two Firmicutes (one *Bacilllus* sp. and one *Paenibacillus* sp.) were also observed to inhibit all strains comprising the ARP. These two genera are well-documented secondary metabolite producers known to produce a plethora of antimicrobial compounds (Pathma et al., 2011; Caulier et al., 2019).

Interestingly, *Paenibacillus* sp. GHS.8.NWYW.5 and *Pseudomonas* sp. GHCE.5.JVZL.12 were the only isolates passing dereplication that were not derived from active layer permafrost. *Paenibacillus* sp. GHS.8.NWYW.5 was isolated from sediments of the Gypsum Hill saline spring (∼8% salts) and *Pseudomonas* sp. GHCE.5.JVZL.12 was isolated from a gypsum cryptoendolith. Our current knowledge of high Arctic saline spring sediments and cryptoendoliths as potential reservoirs of secondary metabolites is very limited and thus this finding serves as an interesting starting point for future studies.

### Two Promising Isolates Inhibit the Growth of Foodborne and Clinically Relevant Pathogens

After the antibiotic activity of all eight isolates that passed the dereplication assay was tested against foodborne and clinically relevant pathogens, *Pseudomonas* sp. AALPS.10.MNAAK.13 and *Paenibacillus* sp. GHS.8.NWYW.5 were identified to have a broad spectrum of antibacterial activity. *Pseudomonas* sp. AALPS.10.MNAAK.13 was found to have the broadest activity against clinically relevant pathogens; inhibiting the growth of both Gram-positive and Gram-negative pathogens including MRSA, *A. baumanii*, MSSA, *E. faecium*, and *E. faecalis. Paenibacillus* sp. GHS.8.NWYW.5 was capable of inhibiting all four foodborne pathogens tested in this study. It displayed inhibitory effects at 37°C and at 5°C against *S. aureus*, *S. enterica*, *L. monocytogenes*, and *E. coli* O157:H7 (Tables 5, 6). The zones of inhibition produced by these Arctic isolates were visually similar to ones produced by the environmental reference strain *P. terrae* NRRL B-30644 which was initially isolated from a Russian poultry production environment and documented to have inhibitory activity against the foodborne pathogen *Campylobacter jejuni* (Lohans et al., 2014). To confirm that the antagonistic activities observed during the initial co-culture assays were due to secreted bacterial biomolecules, promising isolates were selected for organic extraction and tested against the parental strains of the ARP. The organic extract of isolates *Pseudomonas* sp. AALPS.10.MNAAK.13, and *Paenibacillus* sp. GHS.8.NWYW.5 exhibited zones of clearance against *E. coli* Δ*bam*Δ*tolC* BW25113. These observations coupled with our detection of secondary metabolite gene clusters within Arctic isolates’ genomes (Table 7), suggest that the inhibitory activities observed in this study arose from secreted antibacterial secondary metabolites. Ongoing testing is taking place to identify the causative agent(s) within the crude extracts and to determine their potency and spectrum of activity.

Interestingly, both of these Arctic isolates displayed antibacterial activity against MRSA and MSSA. MRSA’s prevalence and the number of deaths it causes yearly qualify this pathogen as a major global public health concern (Martin et al., 2019), and antibacterial activity against MRSA is therefore of particular interest. The recent discovery of malacidin (effective against MRSA and other Gram-positive pathogens) illustrates how natural products from soil-dwelling bacteria continue to represent promising drug leads for endemic antibiotic resistant pathogens of the 21st century (Hover et al., 2018). The secondary metabolites expressed by the Arctic isolates surveyed herein will be further characterized in future studies to determine if they have any chemotherapeutic potential.

The genome of *Paenibacillus* sp. GHS.8.NWYW.5 was found to 7 BGCs displaying low homology with clusters of its respective type strains of the same species. Conversely, all clusters detected within the genome of *Pseudomonas* sp. AALPS.10.MNAAK.13 displayed high homology when aligned with the type strain genome sequence. The genome of *Paenibacillus* sp. GHS.8.NWYW.5 featured the greatest number of total BGCs with five NRP clusters displaying low homology to its corresponding type strain genome. The type strain of this isolate, *P. terrae* NRRL B-30644 has been shown to express paenicidins and tridecaptin A_1_ (Lohans et al., 2014). Clusters encoding both paenicidin and tridecaptin were identified within the genome of *Paenibacillus* sp. GHS.8.NWYW.5, however, it was also found to encode five NRP clusters with low homology to the type strain genome. Follow-up experiments such as genome-wide transposon mutagenesis or targeted cloning and exogenous expression of specific clusters are required to determine which cluster(s) are responsible for the observed antibiotic activity exhibited by these isolates.

### Arctic Isolates That Passed the ARP Are Capable of Growth at Refrigeration Temperatures

All isolates that passed the dereplication assay (except for *Pseudomonas* sp. GHCE.5.JVZL.12) were capable of growing at temperatures ≤ 0°C within 20 days of incubation and grew more quickly at room temperature. Only two isolates (*Paenibacillus* sp. GHS.8.NWYW.5 and *Pseudomonas* sp. GHCE.5.JVZL.12) were capable of growing at 37°C. This observation suggests that these isolates are eurypsychrophiles which are typically characterized by their tolerance to subzero temperatures and display optimal growth at ∼20°C (Raymond-Bouchard et al., 2018). Previous studies conducted with similar soil samples from the MARS site have led to the isolation of eurypsychrophillic strains including *Planococcus halocryophilus* Or1 that is capable of growth at –15°C (Mykytczuk et al., 2016). The initial incubation of the bulk soil plates was performed at room temperature precluded the isolation of stenopsychrophiles that would have required lower cultivation temperatures (Raymond-Bouchard et al., 2018).

The antibacterial activity of *Paenibacillus* sp. GHS.8.NWYW.5 against foodborne pathogens at common household refrigeration temperatures makes it a promising candidate for biotechnological applications in food-safety. The use of cold-adapted environmental strains as biopreservatives intended to inhibit the growth of foodborne pathogens within refrigerated commodities have been previously developed (Rovira and Melero, 2018). For example, certain strains of bacteriocin-producing *Carnobacteria* spp. isolated from natural environments have been used to limit the growth of pathogens within preserved seafood products (Wiernasz et al., 2017). The isolates identified in this study that were capable of subzero growth and displayed antibacterial activity at refrigeration temperatures could serve as interesting starting points for future food-safety applications.

## Conclusion

The results of our bioprospecting demonstrate how crowdsourced classical microbial cultivation and cryo-iPlate cultivation contribute to a culture-dependent workflow that can lead to the identification of Arctic bacteria capable of antibacterial activity. First results suggest that the habitats within the vicinity MARS on Axel Heiberg Island harbor promising cold-adapted bacteria with antibacterial activities against both foodborne and clinically significant pathogens. Further investigation will be required to identify the causative agents responsible for the observed antibacterial activities and to explore potential clinical and food-safety applications.

## Data Availability

The raw data supporting the conclusions of this manuscript will be made available by the authors, without undue reservation, to any qualified researcher.

## Author Contributions

EM carried out field work, lab work, analysis, and writing. MO carried out analysis, participated in experimental design, and contributed significantly to writing. TL and GM conducted the dereplication assay and screening against clinical pathogens. EH conducted the dereplication assay and participated in isolating and screening cryo-iPlate strains. JH and BB conducted whole genome sequencing and assembly. GA and OB-H assisted in isolating and screening cryo-iPlate strains. RL and DN advised in experimental design and writing. SG coordinated the MIMM212 undergraduate crowdsourcing laboratory course, and advised and contributed significantly to writing and analysis. LW supervised the project and significantly contributed to project design, writing and analysis.

## Conflict of Interest Statement

The authors declare that the research was conducted in the absence of any commercial or financial relationships that could be construed as a potential conflict of interest.

## Footnotes

1 https://nucleobytes.com/
