# Supplementary material for: Culture-Dependent Bioprospecting of Bacterial Isolates From the Canadian High Arctic Displaying Antibacterial Activity
Source: Front Microbiol. 2019 Aug 9;10:1836. doi: 10.3389/fmicb.2019.01836 (PMC6696727; doi:10.3389/fmicb.2019.01836)
Supplement: Supplementary file 1 [file Data_Sheet_1.docx]

Supplementary Figures and tables

Table S1. ESKAPE relatives used to screen for antibacterial activity

| ESKAPE pathogen | Safe ESKAPE relative | ATCC |
| --- | --- | --- |
| *Enterococcus faecium* | *Enterococcus raffinosus* | 49464 |
| *Staphylococcus aureus* | *Staphylococcus epidermidis* | 14990 |
| *Klebsiella pneumoniae* | *Escherichia coli* | 11775 |
| *Acinetobacter baumannii* | *Acinetobacter baylyi* | 33305 |
| *Pseudomonas aeruginosa* | *Pseudomonas putida* | 12633 |
| *Enterobacter spp.* | *Enterobacter aerogenes* | 51697 |

Table S2. Antibiotic resistant strains comprising the dereplication platform used in this study [[13](#_ENREF_13)].

| **Antibiotic class** | **Antibiotic** | **Resistance Gene** | **Plasmid** | **Promoter** | **Parent *E. coli* strain** |
| --- | --- | --- | --- | --- | --- |
| Aminoglycosides | Streptomycin | *aph(3’)-Ia* | pGDP3 | P_bla_ | Δ*bam*Δ*tolC* BW25113 |
|  | 2-Deoxystreptamine | *rmtB* | pGDP3 | P_bla_ | Δ*bam*Δ*tolC* BW25113 |
|  | Apramycin | *apmA* | pGDP3 | P_bla_ | Δ*bam*Δ*tolC* BW25113 |
|  | Spectinomycin | *Aph(9)-Ia* | pGDP3 | P_bla_ | Δ*bam*Δ*tolC* BW25113 |
| Β-lactams | Penicillin | *NDM-1* | pGDP1 | P_bla_ | Δ*bam*Δ*tolC* BW25113 |
|  | Cephalosporin | *NDM-1* | pGDP1 | P_bla_ | Δ*bam*Δ*tolC* BW25113 |
|  | Carbapenam | *NDM-1* | pGDP1 | P_bla_ | Δ*bam*Δ*tolC* BW25113 |
| Lincosamides | Lincosamides | *ermC* | pGDP4 | P_lac_ | Δ*bam*Δ*tolC* BW25113 |
| Macrolides | Macrolides | *ermC* | pGDP4 | P_lac_ | Δ*bam*Δ*tolC* BW25113 |
| Type B Streptogramins | Type B Streptogramins | *ermC* | pGDP4 | P_lac_ | Δ*bam*Δ*tolC* BW25113 |
| Type A Streptogramins | Type A Streptogramins | *vatD* | pGDP3 | P_bla_ | Δ*bam*Δ*tolC* BW25113 |
| Streptothricin | Streptothricin | *STAT* | pGDP1 | P_bla_ | Δ*bam*Δ*tolC* BW25113 |
| Tetracyclines | Tetracycline | *tet(A)* | pGDP4 | P_lac_ | Δ*bam*Δ*tolC* BW25113 |
| Chloramphenicols | Chloramphenicols | *CAT* | pGDP3 | P_bla_ | Δ*bam*Δ*tolC* BW25113 |
| Fosfomycins | Fosfomycins | *fosA* | pGDP1 | P_bla_ | Δ*bam*Δ*tolC* BW25113 |
| Rifamycins | Rifamycins | *arr* | pGDP3 | P_bla_ | Δ*bam*Δ*tolC* BW25113 |
| Polymyxins | Polymyxins | *MCR-1* | pGDP1 | P_bla_ | Wild-type BW25113 |
| Echinomycins | Echinomycins | *uvrA* | pGDP1 | P_bla_ | Δ*bam*Δ*tolC* BW25113 |
| Sideromycins | Albomycin | *fhuB mutant* | - | - | Δ*bam*Δ*tolC* BW25113 |
| Tuberactinomycins | Viomycin | *vph* | pGDP1 | P_bla_ | Δ*bam*Δ*tolC* BW25113 |
|  |  |  |  |  |  |

**Table S3**. **Assessing growth of Arctic isolates that inihibited the ARP at various temperatures.**

| **Isolate name** | **Closest matching taxon** | **Closest matching strain** | **37*°*C**  **t= 48hrs** | **25*°*C**  **t= 48hrs** | **10*°*C**  **t= 20 days** | **5*°*C**  **t= 20 days** | **0*°*C**  **t= 20 days** | **-5*°*C**  **t= 20 days** |
| --- | --- | --- | --- | --- | --- | --- | --- | --- |
| GHHS.3.LBZX.4 | *Flavobacterium panaciterrae* | DCY69(T) |  | **+** | **+** | **+** | **+** |  |
| GHS.8.NWYW.5 | *Paenibacillus terrae* | AM141(T) | **+** | **+** | **+** | **+** | **+** | **+** |
| AALPS.10.MNAAK.13 | *Pseudomonas prosekii* | LMG 26867 |  | **+** | **+** | **+** | **+** | **+** |
| MAL.10.WYTK.25 | *Pseudomonas extremaustralis* | 14-3(T) |  | **+** | **+** | **+** | **+** | **+** |
| GHCE.5.JVZL.12 | *Pseudomonas fluorescens* | DSM 50090(T) | **+** | **+** | **+** | **+** |  |  |
| AALPS.4.MSMB.5 | *Pseudomonas mandelii* | NBRC 103147(T) |  | **+** | **+** | **+** | **+** | **+** |
| MAL.4.ABES.21 | *Pseudomonas frederikbergensis* | JAJ28(T) |  | **+** | **+** | **+** | **+** |  |
| C11E23 | *Bacillus tequilensis* | KCTC 13622(T) |  | **+** | **+** | **+** | **+** | **+** |
| (+) Visible growth |  |  |  |  |  |  |  |  |

**Table S4. Genomic sequencing statistics**

| **Isolate** | **Contigs** | **Scaffolds** | **Genome Size** | **Longest Scaffold** | **N50** | **Raw reads** | **Error-corrected Reads** | **% reads passing Error-correction** | **Raw nucleotides** | **Error-corrected nucleotides** | **% nt passing Error-correction** | **Raw coverage** | **Median coverage** | **10th percentile coverage** | **Bases**  **>= Q40** |
| --- | --- | --- | --- | --- | --- | --- | --- | --- | --- | --- | --- | --- | --- | --- | --- |
| GHHS.3.LBZX.4 | 69 | 53 | 6116726 | 806308 | 501802 | 1145402 | 1129059 | 98.57 | 320233758 | 267677262 | 83.59 | 43.76 | 51 | 39 | 6112985 |
| GHS.8.NWYW.5 | 73 | 55 | 5662149 | 609831 | 274348 | 3038748 | 2928247 | 96.36 | 889616513 | 647752279 | 72.81 | 114.4 | 149 | 126 | 5660236 |
| AALPS.10.MNAAK.13 | 36 | 32 | 6090438 | 798849 | 523870 | 1272094 | 1192269 | 93.72 | 338765113 | 248848688 | 73.46 | 40.86 | 50 | 38 | 6084513 |
| MAL.10.WYTK.25 | 62 | 60 | 6826261 | 442973 | 256343 | 676206 | 665502 | 98.42 | 179401430 | 138717033 | 77.32 | 20.32 | 25 | 16 | 6807762 |
| GHCE.5.JVZL.12 | 48 | 44 | 4977715 | 625630 | 423958 | 1259932 | 1207488 | 95.84 | 332870865 | 244986609 | 73.6 | 49.22 | 62 | 48 | 4974582 |
| AALPS.4.MSMB.5 | 79 | 60 | 6323405 | 523195 | 223859 | 1837634 | 1800987 | 98.01 | 506142769 | 380213292 | 75.12 | 60.13 | 76 | 60 | 6321054 |
| MAL.4.ABES.21 | 23 | 21 | 6812670 | 1022152 | 638679 | 972954 | 945125 | 97.14 | 268813615 | 194890214 | 72.5 | 28.61 | 37 | 27 | 6809466 |
| C11E23 | 25 | 18 | 4271209 | 2223793 | 2223793 | 1675208 | 1637939 | 97.78 | 485678175 | 416677039 | 85.79 | 97.55 | 110 | 90 | 4265909 |


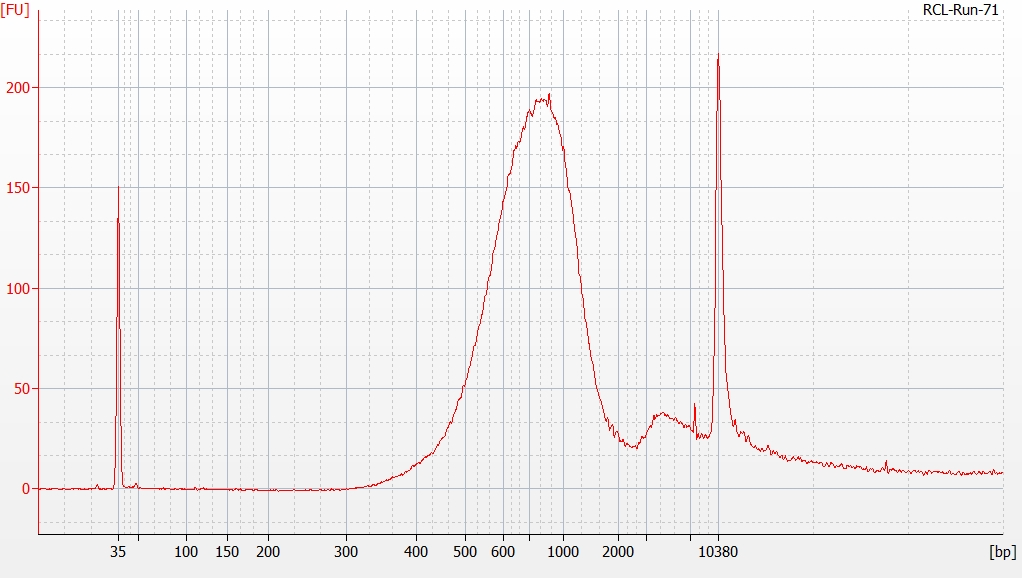


**Figure S1. High sensitivity DNA electropherogram of the pooled Illumina MiSeq library** The electropherogram was obtained from an Agilent High Sensitivity DNA Chip on a Agilent 2100 Bioanalyzer.. The x-axis represents DNA fragment size, the y-axis represents fluorescence units.
